# *CHEK2* germline variants in B-cell precursor acute lymphoblastic leukemia: findings in Mexican pediatric patients

**DOI:** 10.3389/fonc.2026.1751793

**Published:** 2026-03-16

**Authors:** Daniel Martínez Anaya, Liliana Fernández Hernández, Marian Valladares Coyotecatl, Ulises Juárez Figueroa, Michael Dean, Luis Juárez Villegas, Marta Zapata Tarrés, Norma López Santiago, Patricia Pérez-Vera

**Affiliations:** 1 Laboratorio de Genética y Cáncer, Instituto Nacional de Pediatría, Mexico City, Mexico; 2 Posgrado en Ciencias Biológicas, Unidad de Posgrado, Universidad Nacional Autónoma de México, Ciudad Universitaria, Mexico City, Mexico; 3 Laboratorio de Biología Molecular, Instituto Nacional de Pediatría, Mexico City, Mexico; 4 Laboratorio de Citogenética, Instituto Nacional de Pediatría, Mexico City, Mexico; 5 Laboratory of Translational Genomics, Division of Cancer Epidemiology and Genetics, National Cancer Institute, Rockville, MD, United States; 6 Servicio de Hemato-Oncología, Hospital Infantil de México Federico Gómez, Mexico City, Mexico; 7 Comisión Coordinadora de Institutos Nacionales de Salud y Hospitales de Alta Especialidad, Mexico City, Mexico; 8 Servicio de Hematología, Instituto Nacional de Pediatría, Mexico City, Mexico

**Keywords:** B-cell precursor acute lymphoblastic leukemia, cancer predisposition, *CHEK2* germline variants, *CHEK2* p.Leu236Pro, germline predisposition, Mexican pediatric patients

## Abstract

**Background:**

Deleterious *CHEK2* germline variants (GVs) are moderate-penetrance risk alleles that predispose individuals to adult-onset neoplasms. However, their association with childhood-onset cancers, such as B-cell precursor acute lymphoblastic leukemia (pre-B ALL), remains unexplored.

**Aim:**

To describe the mutational profile of *CHEK2* GVs in a cohort of Mexican children diagnosed with pre-B ALL and review the mutational landscape of *CHEK2* GVs in children with pre-B ALL.

**Methods:**

Next-generation exome sequencing was performed on 73 Mexican children with pre-B ALL. Clinical and genetic features of *CHEK2* GVs carriers have been described. Associations between *CHEK2* GVs and predisposition to pre–B ALL were evaluated using the MCPS population datasets as control groups. In addition, a literature review was conducted to investigate the potential link between *CHEK2* germline variants and pre-B ALL. Finally, an in silico analysis was performed using bioinformatic tools and protein modeling to predict the functional and structural effects of these variants.

**Results:**

*CHEK2* GVs were identified in four patients with high-risk pre-B ALL, two carried likely pathogenic variants (2.7%) and two carried variants of uncertain significance (2.7%). Three of these patients died due to disease progression, and two had a family history consistent with the *CHEK2* cancer predisposition spectrum. Two unrelated cases carried the likely pathogenic *CHEK2* p.Leu236Pro variant. When compared with the Indigenous Mexican stratum of the MCPS database, this variant was associated with pre–B ALL predisposition (unadjusted OR, 5.48; 95% CI, 1.34–22.37). Including previously reported cases, a total of 34 individuals with pre–B ALL carrying 20 distinct *CHEK2* GVs were identified in the literature. Most variants were population-specific and predicted to impair protein function or structural stability. Structural modeling suggested that the recurrent *CHEK2* p.Leu236Pro variant may introduce steric hindrance affecting protein dimerization.

**Conclusions:**

Our findings and those described in the literature suggest that *CHEK2* may play a role in the germline origin of childhood pre-B ALL in specific populations. However, this study provides preliminary evidence of pre-B ALL predisposition in Mexican children with *CHEK2* GVs that needs replication in a larger cohort to obtain accurate estimations.

## 1 Background

The *CHEK2* gene is located at 22q12.1 and encodes checkpoint kinase 2 (CHK2), a critical cell cycle checkpoint regulator and putative tumor-suppressor protein. CHK2 is activated by ATM protein through phosphorylation in response to DNA damage and replication blocks (1). Once activated, CHK2 inhibits CDC25C phosphatase, which impedes entry into mitosis. Additionally, CHK2 stabilizes the tumor suppressor protein p53, promoting cell cycle arrest in the G1 phase. CHK2 interacts with and phosphorylates BRCA1, modulating its function through homologous recombination-based DNA repair and enabling survival after DNA damage (1).

Deleterious *CHEK2* germline variants (GVs) are associated with cancer predisposition syndromes involving adult-onset malignancies. These syndromes include familial colorectal cancer Type X (ORPHA:440437), familial prostate cancer (ORPHA:1331), osteosarcoma (ORPHA:668), hereditary breast and/or ovarian cancer syndrome (ORPHA:145), and Li-Fraumeni syndrome (ORPHA:524). Nevertheless, the role of *CHEK2* deleterious GVs in the predisposition to hematological malignancies (HMs) remains unclear. Currently, small single-center studies have recognized some *CHEK2* mutations, such as p.Ile157Thr and p. Ser428Phe, which are enriched in patients with myeloid leukemia and non-Hodgkin lymphoma (2, 3). Nonetheless, the role of deleterious *CHEK2* GVs in the predisposition to childhood-onset HMs has not been thoroughly investigated in Mexican individuals.

B-cell precursor acute lymphoblastic leukemia (pre-B ALL) is the most common childhood malignancy worldwide and a public health issue in Mexico (4). It is characterized by the malignant proliferation of B-cell progenitors that are arrested at an early stage of cellular differentiation (5). While most pre-B ALL cases in children occur sporadically, recent studies have revealed that approximately 8.3–11% of cases arise due to deleterious GVs involving cancer predisposition genes (6, 7). These variants can be high-penetrance alleles for pre-B ALL if they involve genes directly associated with leukemogenesis, such as *IKZF1* or *PAX5*, or moderate-penetrance alleles if they involve genes expressed ubiquitously (such as *CHEK2)* and predispose individuals to cancer syndromes with a wide range of tumors, in which pre-B ALL can be a sporadic neoplastic manifestation. Therefore, genetic counseling and personalized oncological management are mandatory to avoid the potential side effects of conventional therapy (8–11).

This study aimed to describe the mutational profile of *CHEK2* GVs in a cohort of 73 Mexican children diagnosed with pre-B ALL using next-generation exome sequencing analysis, targeting cancer predisposition genes. The study also aimed to review the mutational landscape of *CHEK2* GVs in children with pre-B ALL, to explore the potential association. Finally, we performed a bioinformatic analysis to predict the functional and structural effects of *CHEK2* GVs and a protein modeling analysis to propose a potential molecular mechanism of pathogenicity for the recurrent variants identified in our study cohort.

## 2 Methods

### 2.1 Study cohort and sample collection

A total of 73 pediatric patients diagnosed with pre-B ALL aged ≤ 18 years of age were enrolled in this study. Patients were recruited consecutively from January 2015 to December 2018 at the National Institute of Pediatrics and Children’s Hospital of Mexico Federico Gómez, both located in Mexico City. Patients or their guardians signed informed assent/consent forms in accordance with the Declaration of Helsinki. The study was approved by the participating institutions’ Research and Ethics Committees (project numbers 067/2014, 019/2016, and 032/2023, and the National Commission of Bioethics registration number CONBIOETICA-09-CEI-025-20161215). Pre-B ALL diagnoses were established through cytomorphology and immunophenotyping (CD10, CD19, CD20, CD34, and CD79).

Genomic DNA was extracted from the bone marrow samples collected at diagnosis using a DNeasy Blood & Tissue kit (Qiagen, Germany). Quality control measures were implemented to ensure DNA integrity and quantity. High-quality DNA was then used for next-generation exome sequencing.

### 2.2 Next generation sequencing analysis and variant classification

A custom-designed exome sequencing panel targeting 42 cancer predisposition genes (**Supplementary Table 1**) was used (7, 11). Genomic DNA from bone marrow samples were used as source of genomic DNA for Next generation exome sequencing using an Illumina HiSeq 2000 platform, according to the manufacturer’s instructions. This molecular approach allowed us to detect SNV/Indel variants; however copy number variants (CNVs) cannot be interrogated by this technique. An in-house script was used for quality control, alignment, calibration, and annotation of variants, followed by analysis following the best practices of Genome Analysis Toolkit. The average sequencing coverage depth was 500X for all target regions. Only variants in regions with a minimum coverage threshold of ≥30X were considered. Regions that reached this threshold were excluded from variant calling. The variant allele fraction (VAF) was calculated by dividing the number of variant-supporting reads by the total number of reads at each variant site.

Variants were manually classified according to the five-tier ACMG/AMP 2015 criteria using Franklin by Genoox database version 80.2. Only variants classified as pathogenic, likely pathogenic or VUS were reported. Variants in cancer predisposition genes were assumed to be potential germline variants if they were present in patients diagnosed with the associated genetic syndrome, and/or exhibited a VAF of ~0.50 or 1.0, ideally corresponding to heterozygous or homozygous state respectively (or >0.30 if the genomic region was affected by CNVs verified by chromosome microarray analysis), based on the Kraft & Godley 2020 guidelines for the identification of potential germline alleles from NGS assay of tumor samples (12).

### 2.3 Chromosome microarray analysis

The patients positive to potential germline variants in *CHEK2* were characterized by chromosome microarray analysis performed in genomic DNA, derived from bone marrow samples. We used a high-resolution array-based comparative genomic hybridization (aCGH) with a 2x 400 K SurePrint G3 Custom CGH Human Genome Microarray (Design ID #21850; Agilent Technologies, Waldbronn, Germany), or a CytoScan 750 K array (Affymetrix, Santa Clara, California, USA). The microarrays were processed according to the manufacturer’s protocol. Regarding to aCGH, the TIF images were extracted using the Agilent Feature Extraction software and the CEL files were analyzed using Cytogenomics v5.3.0.14 software. The CGH v2 ADM-2 algorithm was applied for variant calling. For SNP arrays, the.CEL files were processed using the Chromosome analysis suite 4.5, and converted to CYCHP files using a single-sample analysis workflow with NetAffx 33.1 (hg19) as the annotation file.

### 2.4 *CHEK2* germinality validation and familial genetic screening

The *CHEK2* potential germline variants identified in this study were confirmed in saliva samples from two patients who consented to germinality validation. Genomic DNA were obtained using the prepIT-L2P kit (DNA Genotek Inc., Ottawa, ON, Canada). Specific oligonucleotides were designed for Sanger sequencing and are available upon request. The reactions were analyzed using an ABI 3730X1 capillary electrophoresis system (Applied Biosystems, Foster City, CA, USA) following the manufacturer’s recommendations. The AB1 files were analyzed using Chromas v2.6.6 software.

We searched for *CHEK2* GVs in the proband’s first-degree relatives, if the family reported a history of cancer and/or consented to the assessment. For genetic screening of the family, Sanger sequencing was performed on DNA obtained from peripheral blood samples.

### 2.5 Statistical analysis

We consulted the frequency of the *CHEK2* GVs variants observed in our study in population databases including GnomAD exomes v2.1 (http://gnomad-sg.org) and the Mexico City Prospective Study (MCPS) (https://rgc-mcps.regeneron.com/home) database (consulted in October 2025). The categorical variables were expressed as frequencies and percentages. A two-tailed Fisher´s exact test was used to compare the differences between the groups.

For associacion analysis between *CHEK2* GVs and pre-B ALL predisposition, a case-control analysis was conducted, comparing the distribution of the variants observed in our study cohort (n=73) with two datasets, considered as control groups, obtained from the MCPS database:

1. The dataset of the entire cohort of Mexican individuals (n=138,200).
2. The stratum classified as “Indigenous Mexican” (n=93,978), was used to address the potential confounding factors related to population structure.

Odds ratios (ORs) with 95% confidence intervals were calculated to estimate the association between *CHEK2* GVs and pre-B ALL. Due to the absence of individual-level covariate data from publicly sourced population controls, it was not possible to perform conventional covariate-adjusted regression models. Consequently, only unadjusted ORs were reported as the primary measure of association. To evaluate the potential impact of unmeasured confounding factors (such as age and sex) on the observed associations, a quantitative bias analysis was conducted using algebraic bias factors, as described by Lash et al. (13). Additionally, E-values were obtained according to the method proposed by VanderWeele and Ding (14), which quantifies the minimum strength of association that an unmeasured confounder would need to have with both *CHEK2* GVs and pre-B ALL predisposition to fully explain the observed associations.

For all comparisons, statistical significance was defined as *p* ≤ 0.05. All analyses were performed using SPSS version 21 (IBM Corp., Chicago, IL, USA).

### 2.6 Literature review and database mining

We searched for cases of pediatric pre-B ALL that were positive for *CHEK2* GVs. We conducted a PubMed literature search restricted to English-language articles, including the following terms: ‘CHEK2’, ‘CHK2’, ‘variants’, ‘Acute Lymphoid Leukemia’, ‘Acute Lymphoblastic Leukemia’, ‘Acute Lymphoid Leukemia’ and ‘Acute Lymphoblastic Leukemia’. Reviews, meta-analyses, and studies on T-cell ALL were excluded. Additionally, genomic databases were mined using the cBioPortal TARGET (2018) Pediatric Acute Lymphoid Leukemia-Phase II dataset and PeCan St. Jude Cloud.

PubMed and database searches will be performed until August 2025. Variants recorded in the literature and databases were classified according to the 2015 ACMG/AMP guidelines. Cases with positive results for likely benign or benign variants were excluded from the study.

### 2.7 In silico prediction analysis

The functional effects of *CHEK2* GVs associated with pre-B ALL were predicted using the SIFT, PolyPhen-2, and Mutation Taster tools. Protein stability changes resulting from point mutations were predicted using I-Mutant 2.0 and MuPRO. The ConSurf and VarSite websites were used for the conservation analysis. Default parameters were used for all in silico predictions. To propose a possible molecular pathogenicity mechanism for the recurrent mutations observed in our study cohort, the three-dimensional X-ray crystal diffraction structures of the CHK2 kinase domain in the active and wild type forms (PDB: 3I6W and 2CN5 respectively) were downloaded from the RCSB Protein Data Bank (https://www.rcsb.org) and used for in silico mutagenesis analysis. This was performed using PyMOL 4.6 software.

## 3 Results

### 3.1 Genetic and clinical features of *CHEK2* GVs carriers

**Figure 1:**
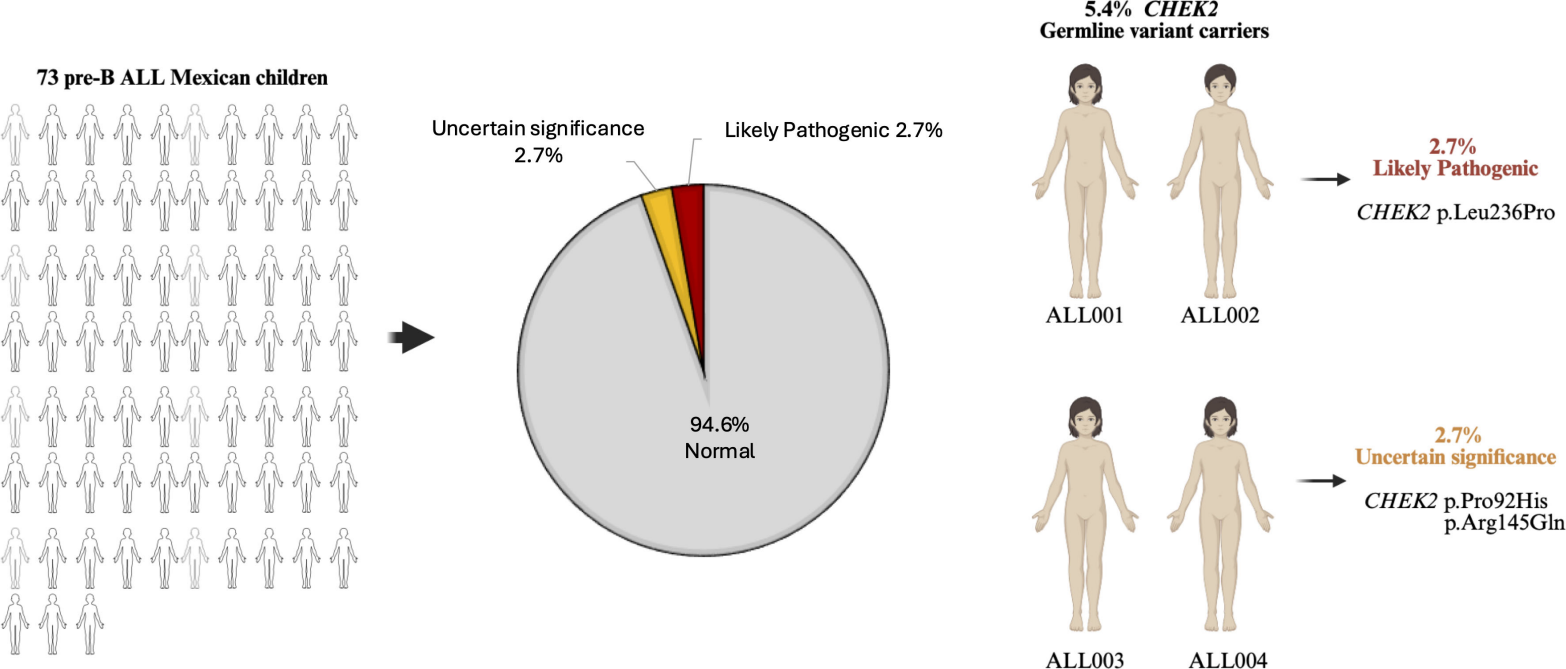
The frequency of *CHEK2* potential germline variants in 73 Mexican children diagnosed with pre-B ALL.

Potential germline *CHEK2* variants were identified in four patients in the study cohort. Two unrelated cases (ALL001 and ALL002) were positive for *CHEK2* p.Leu236Pro likely pathogenic variant (2.7%). The remaining two cases (ALL003 and ALL004) were positive for variants of uncertain significance (2.7%), including the *CHEK2* p.Pro92His variant observed in patient ALL003. The *CHEK2*p. Arg145Gln variant was observed in patient ALL004 (**Figure 1** and **Supplementary Table 2**). No other pathogenic, likely pathogenic, or uncertain significance variants involving the *CHEK2* gene were observed in the study population.

The germline orthogonal validation was consented and tested positive for two patients, the one with the likely pathogenic *CHEK2* p.Leu236Pro and the one with the VUS *CHEK2* p.Pro92His (**Supplementary Table 2** and **Supplementary Figure 1**). The remaining two patients did not consent to be tested for germline status. Nevertheless, the germline origin of these two patients was inferred based on a VAF of ~0.5 (excluding CNVs that could alter this measurement). In addition, one of the patients had a positive family history of cancer compatible with the *CHEK2-*cancer predisposition spectrum (**Supplementary Table 2** and **Supplementary Figure 1**). Furthermore, the three *CHEK2* variants identified as potentially germline in this study were previously recorded in the ClinVar database as non-polymorphic variants associated with *CHEK2*-related cancer predisposition syndromes. Therefore, there is substantial evidence suggesting that the *CHEK2* variants observed in the four patients in this study cohort were of germline origin.

No other potential germline pathogenic variants were identified in the four patients mentioned, except for patient ALL002, who was positive for the potential germline pathogenic variant *PTPN11*(NM_002834.5):c.172A>T(p.Asn58Tyr) (**Supplementary Table 2**).

In terms of general clinical features, carriers of *CHEK2* GVs were diagnosed with pre-B ALL as their first cancer. They were classified as high-risk based on the National Cancer Institute (NCI) criteria. Most patients were female (4:1). The median age at diagnosis was 12.75 years (range 8-16) and the median white blood cell count was 169.350x10^3^/µL (range 400-467.700). Three patients were classified as B-Other because they were negative for classical genetic abnormalities, and one was classified as the Ph-like molecular subtype because of the presence of the *IGH::CRLF2* gene fusion. Most patients were treated with standard chemotherapeutic regimens; however, three died because of disease progression. Finally, two patients had a family history of cancer consistent with the *CHEK2* cancer predisposition spectrum (**Supplementary Table 2**).

#### 3.1.1 Clinical features of *CHEK2* p.Leu236Pro carriers

Patient ALL001 was a 13-year-old female diagnosed with pre-B ALL in September 2017. A summary of the blood analysis results is provided in **Supplementary Table 2**. The immunophenotype of the leukemic blast was consistent with precursor B-cells, and routine genetic analysis revealed *CRLF2:: IGH* gene fusion. Based on these results and the NCI criteria, the patient was stratified as a high-risk pre-B ALL patient. She was treated with the Berlin-Frankfurt-Münster 90 Phase B protocol; however, she discontinued treatment for seven weeks between the first high-risk blocks HR2 and HR3. Six months after initiating induction therapy with HR3 (March 2018), she experienced an early bone marrow relapse. She initiated a second line of treatment according to the Children’s Oncology Group AALL1331 protocol. After the third block, she experienced chemotherapy-related myelotoxicity and received a red blood cell transfusion and platelet apheresis. Three months later (June 2018), she presented with minimal residual disease of 0.01%. She received her last chemotherapy five months later (November 2018). One month later, she experienced a second relapse in the bone marrow and the first in the central nervous system (December 2018). Two weeks later (January 2019), she was considered to be out of curative treatment and received palliative care. The patient died one month later (February 2019).

**Figure 2:**
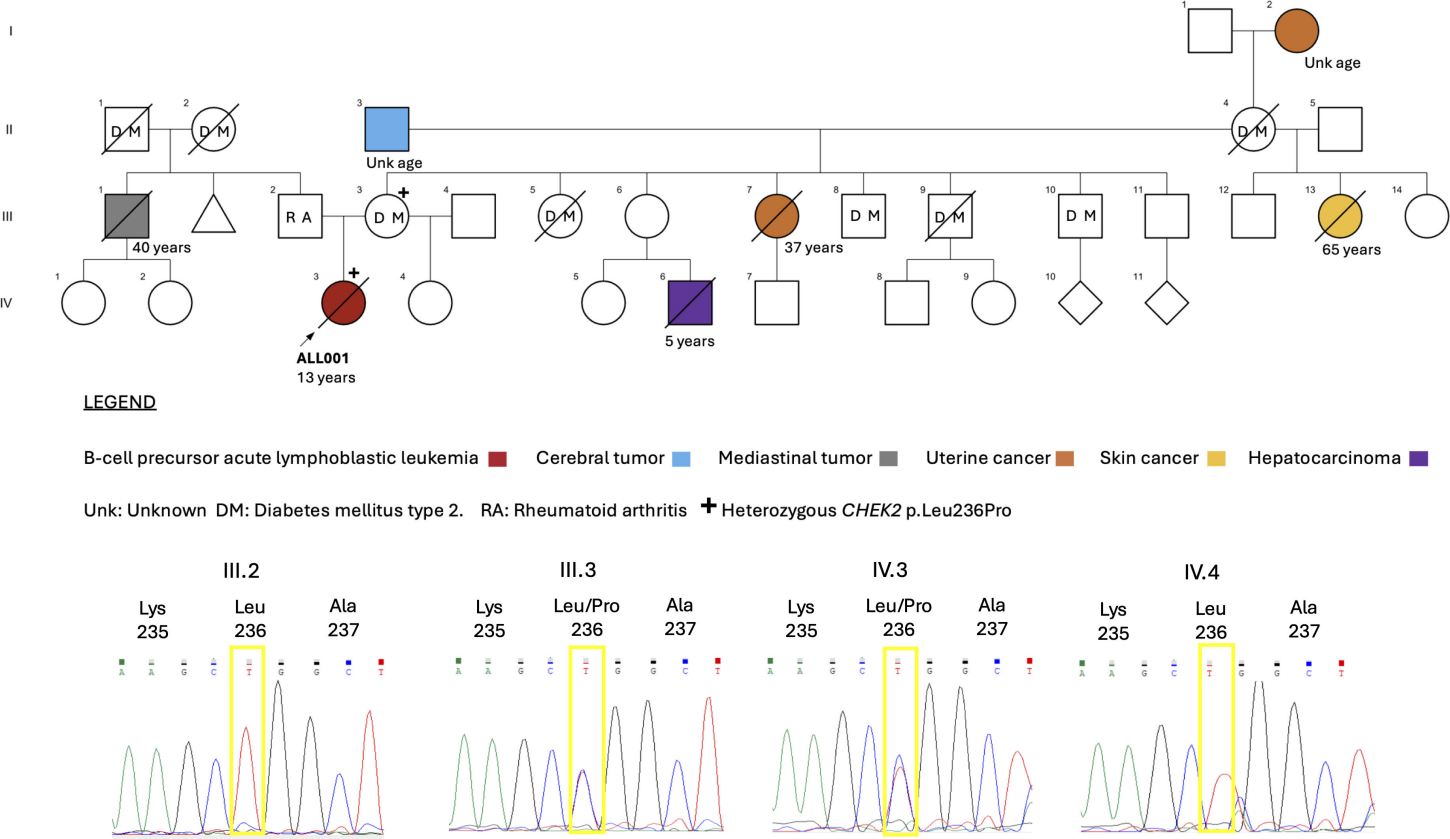
The ALL001 family pedigree and genetic screening for first-degree relatives. Different types of cancer occur on the maternal side. The proband (IV.3) (indicated by the arrow) and her mother (III.3) were found to be heterozygous carriers for the CHEK2 p.Leu236Pro variant. The father (III.2) and the maternal half-sister (IV.4) were negative for the variant.

The patient ALL001 did not showed congenital anomalies and had a family history of cancer that was consistent with the *CHEK2-*cancer predisposition spectrum. The pedigree analysis shows that she was the first child of a mother with type 2 diabetes mellitus and a father with rheumatoid arthritis (**Figure 2**). She has an apparently healthy half-sister on her mother’s side of the family. Her maternal aunt died of uterine cancer with lung metastases at 37 years of age. A cousin had died of hepatocellular carcinoma at the age of five years. The patient’s grandfather died of a brain tumor, her great-grandmother died of uterine cancer, and her half-aunt on her mother’s side died of an unspecified skin cancer. In her paternal family history, her uncle died of a mediastinal tumor at the age of 40 years. The proband´s non-first degree relatives were not tested for *CHEK2* germline mutations. However, familial genetic screening of the proband’s first-degree relatives revealed a maternal segregation of the *CHEK2* p. Leu236Pro variant. Her father and half-sister were negative for the variant (**Figure 2**).

Patient ALL002, a 14-year-old male, was diagnosed with pre-B ALL in June 2017. His blood count are presented in **Supplementary Table 2**. The immunophenotype of the leukemic blasts was consistent with that of precursor B-cells. The patient was negative for classical ALL genetic abnormalities and was classified as B-other. Based on these results, the NCI stratified the patient as a high-risk B-ALL patient. Ten days after admission for treatment, the patient died of septic shock caused by *E. coli* infection. His parents did not report any family members with a history of cancer, and no congenital anomalies were documented.

#### 3.1.2 Clinical features of *CHEK2* uncertain significance variant carriers

Patient ALL003 was a 13-year-old female who was diagnosed with pre-B ALL in January 2014. A detailed report of the blood analysis is provided in **Supplementary Table 2**. The immunophenotype of the leukemic blast cells was consistent with that of precursor B cells. Routine genetic analysis revealed no classical genetic abnormalities associated with ALL. Based on these results and the NCI criteria, the patient was categorized as a high-risk B-ALL patient. The patient was treated with the St. Jude XII-B protocol. Fourteen months later (March 2015), she received an intrathecal therapy. Subsequently, she was diagnosed with brain and pulmonary aspergillosis and cytomegalovirus infections. She was deemed ineligible for curative treatment and received palliative care instead. She died two months later (April, 2015). Her parents did not report any family history of cancer and congenital anomalies were absent.

Patient ALL004 was a 16-year-old female diagnosed with pre-B ALL in September 2016. The results of the blood analysis are presented in **Supplementary Table 2**. The immunophenotype of the leukemic blast was consistent with precursor B cells. The patient was negative for classical ALL genetic abnormalities and was classified as B-other type. Based on these results and those of the NCI, she was categorized as a high-risk B-ALL patient. Details regarding the treatment and outcomes were unavailable. The presence of congenital anomalies were not documented. The parents reported a paternal history of breast and gastric cancer, however, they did not consent to genetic screening.

### 3.2 *CHEK2* GVs population allele frequencies and pre-B ALL associations

Regarding to allele frequencies comparisons, the *CHEK2* p.Leu236Pro likely pathogenic variant was identified in two patients in this study cohort. According to the exomes of genomAD, this variant was present at a frequency of 0.034% in 251,368 individuals, corresponding to 87 allele counts in the American subpopulation. The remaining subpopulations were negative for this variant. The MCPS database, which comprises 141,046 exomes from individuals in Mexico City, revealed a frequency of 0.03%. However, when considering the “Indigenous Mexican” subset, the frequency increased to 0.05%. The variant was absent from the European and African subsets.

The *CHEK2* p.Pro92His variant of uncertain significance is a rare allele that was not observed in the population databases used in this study. The *CHEK2* p.Arg145Gln variant is also of uncertain significance in this study. This is a very rare allele, observed at a frequency of 0.0016%, corresponding to four allele counts in the European non-Finnish subpopulation of 251,292 individuals in the gnomAD exome database. According to the MCPS database, this variant is present at a frequency of 7x10^-6^ % in individuals from the ‘European’ subset.

Due to the heterogeneous population structure of GnomAD, only the MCPS population subsets were used as controls in the association analysis. The results are presented in **Supplementary Table 3**. The association study could not be performed for the *CHEK2* variants of uncertain significance because each variant was present in only one patient. Particularly the *CHEK2* p.Pro92His variant was absent from the control datasets.

A statistically significant association was observed between the *CHEK2* p.Leu236Pro variant and predisposition to pre-B ALL when compared with the MCPS control groups. The unadjusted OR for the MCPS-ALL comparison was 8.07 (95% CI, 1.97–32.96). Restricting the analysis to the Indigenous Mexican (IMX) stratum resulted in a diminished unadjusted OR of 5.48 (95% CI, 1.34–22.37), but the association remained statistically significant.

Quantitative bias analysis indicated that imbalances in the age and sex distributions between cases and controls would have a limited impact on the observed associations under bias scenarios considered to be plausible (see **Supplementary Table 4**). Consistent with these findings, the E-value was 10.43 for the point estimate and 2.01 for the lower bound of the confidence interval in the MCPS-IMX restricted analysis (see **Supplementary Table 5**). This suggests that an unmeasured confounder would need to be associated with both *CHEK2* p.Leu236Pro carrier status and pre-B ALL predisposition with a risk ratio of at least this magnitude to fully explain the observed association.

### 3.3 Literature review and database mining

A total of thirty-four pediatric patients with pre-B ALL and *CHEK2* GVs classified as pathogenic, likely pathogenic, or of uncertain significance were identified through a review of the literature, database mining, and considering the four cases described in this study (2, 15–24). These patients were male and female children with diverse pre-B ALL genetic subtypes. The variants were transmitted by either the mother or father, and some cases exhibited a family history of *CHEK2*-associated cancers. (**Supplementary Table 6**).

**Figure 3:**
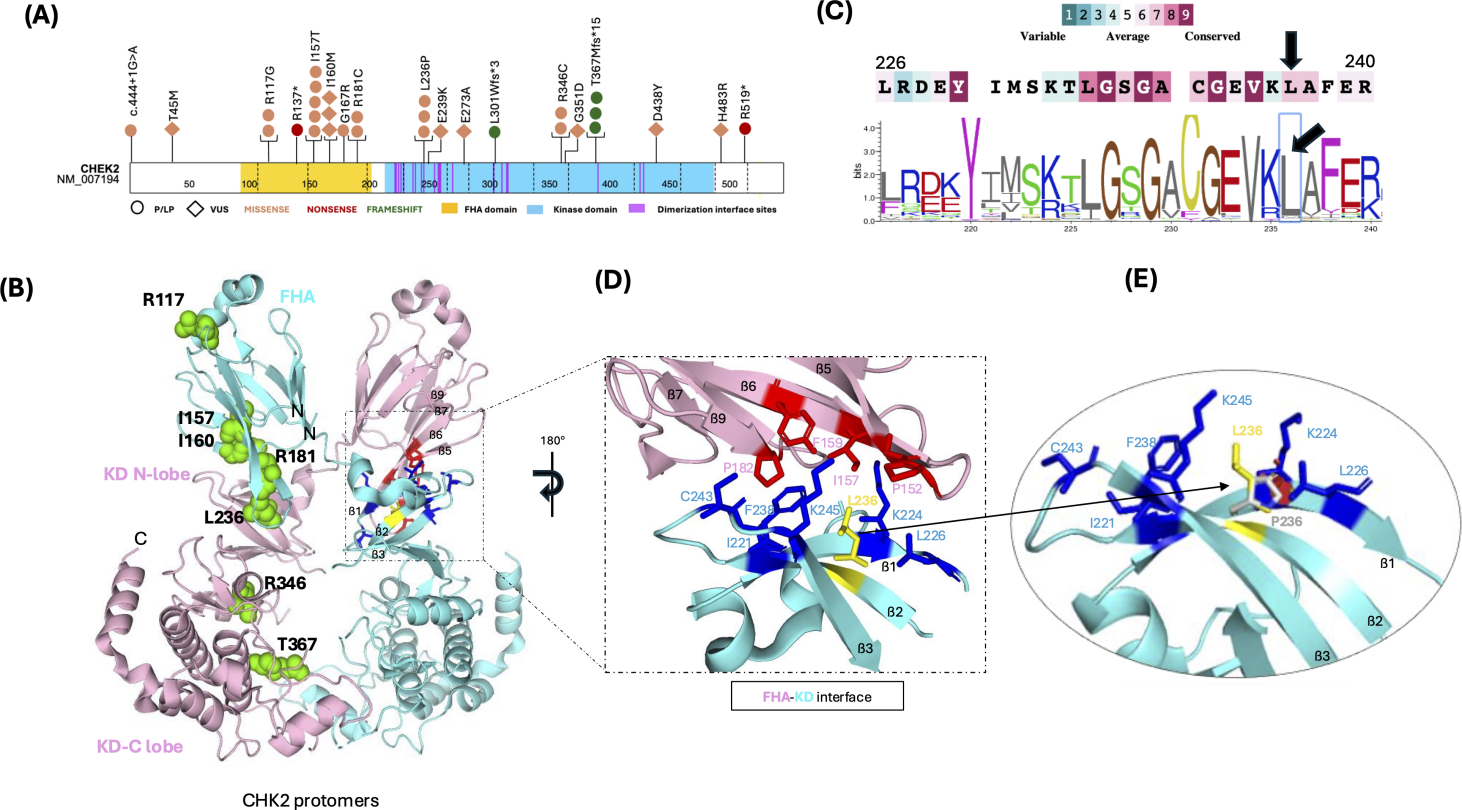
Mutational spectrum of *CHEK2* deleterious germline variants in patients with pre-B ALL and molecular pathogenic mechanism of the Leu236Pro variant. **(A)** Schematic representation of the protein domain location of the residues affected by deleterious germline mutations observed in pre-B ALL. **(B)** Conservation of amino acids around Leu236 (black arrows). The sequence was colored according to the conservation level. The largest letters represent the most conserved amino acids at that site (see below). **(C)** Low-resolution 3D X-ray crystal diffraction structure of CHK2 protomers (blue and pink structures) (PDB: 3I6W). The green spheres represent the residues that are most frequently affected in patients with preB-ALL (left structure). The dimerization interface is enclosed within the rectangle. The amino acid residues that participate in the hydrophobic patch are dark-colored. The Leu236 residue is highlighted in yellow (right structure). **(D)** A close-up view of the intramolecular interface between the FHA (pink) and kinase (blue) domains of the two protomers. The amino acids that comprise the hydrophobic path are shown as dark-colored sticks, and the Leu236 residue is highlighted in yellow. **(E)** High-resolution 3D X-ray crystal diffraction structure of the CHK2 kinase domain (PDB: 2CN5). It shows the steric hindrance (red polygons) between Pro236 and neighboring residues of the hydrophobic patch.

These patients exhibited a mutational spectrum comprising 20 different missense, nonsense, and frameshift mutations distributed along the CHK2 protein (**Figure 3A**). The most frequent mutations involved the following residues: Arg117, Ile157, Ile160, Arg181, Leu236, Arg346, and Thr367. These residues are located in the FHA and kinase domains (**Figure 3B**).

### 3.4 In silico prediction analysis

Most of the 20 different *CHEK2* GVs mutations reported in patients with pre-B ALL were recognized as deleterious changes that decreased the protein’s structural stability, as predicted by at least one prediction tool. Most of these mutations involve highly conserved residues. Interestingly, most of the mutations observed in these patients have already been characterized as having deleterious functional effects using different experimental approaches (**Supplementary Table 6**).

Regarding the recurrent *CHEK2* p.Leu236Pro variant observed in this study, the substitution involves a conserved residue located in the kinase domain at the dimerization interface region. This residue is part of a hydrophobic patch formed by the side chains of the neighboring hydrophobic amino acids. These amino acids establish van der Waals contacts between the FHA domain and the N-lobe of the kinase domain, allowing dimerization of the CHK2 protomers. Protein modeling analysis revealed that the Leu236Pro mutation replaces an aliphatic side chain with a cyclic one. The two possible rotamers of Pro236 induce steric hindrance with the aliphatic β1-Lys224. This residue is also part of a hydrophobic patch. Consequently, this mutation may disrupt the hydrophobic environment necessary for CHK2 dimerization and function (**Figures 3B–E**).

## 4 Discussion

This study aimed to describe the mutational profile of *CHEK2* GVs in a cohort of 73 Mexican children diagnosed with pre-B ALL. Four patients were identified as positive for *CHEK2* likely pathogenic or of uncertain significance potential germline variants. Although there is solid evidence for a germline origin, the germline etiology of two patients relies solely on VAF values and a familial history of cancer. This represents an important limitation of the study. Notably, the *CHEK2* variants identified as germline were recognized by bioinformatic predictors as deleterious changes affecting protein function and structural stability.

Deleterious germline variants of the *CHEK2* gene have been associated with adult-onset cancer predisposition syndromes (1, 25). However, their association with the presentation of childhood cancer remains unclear. To address this, Wagener et al. (15) examined the prevalence of *CHEK2* variants in an unselected cohort of 418 children diagnosed with cancer, as well as in their parents. They reported that pathogenic *CHEK2* germline variants were detected in 7.7% of patients with pediatric cancer. Approximately 20% of these variants were experimentally characterized as deleterious changes, with a significant impact on protein expression or phosphorylation in response to irradiation-induced DNA damage. Interestingly, all carriers of deleterious germline variants developed HMs, with pre-B ALL being the most common (83%) (15). These findings suggest that deleterious *CHEK2* GVs may act as predisposing alleles for childhood cancer, particularly pre-B ALL.

Accordingly, we report two unrelated Mexican patients who were positive for the same *CHEK2* p.Leu236Pro variant, which was detected in 2.7% of 73 Mexican children with pre-B ALL. Interestingly, the p.Leu236Pro mutation was absent from the European and African subsets. It has only been reported in the American subset of the population of the gnomAD database, and in the Indigenous Mexican subset of the MCPS database. This suggests a population-specific enrichment. The *CHEK2* p.Leu236Pro variant has recently been associated with cancer predisposition in Mexican individuals. A total of 5,759 adult patients suspected of having hereditary cancer were analyzed, and the variant was present in 58 patients (1%). Notably, the variant carriers exhibited a geographical clustering pattern, with 81% of the patients originating from a central region of Mexico. Therefore, the authors consider that this variant may be a founder mutation (26).

Consistent with this hypothesis, other *CHEK2* deleterious GVs also exhibited population-specific enrichment. The variant of uncertain significance, *CHEK2* p.Arg145Gln, was observed in the European or European non-Finish subpopulations of the gnomAD and MCPS databases. In addition, Stubbins et al. (2) studied a cohort of 80 individuals with *CHEK2* GVs, most of whom were of European descent. The p.Ile157Thr mutation was the most common variant (55%), and this allele was commonly associated with Polish ancestry. Notably, 77% of these patients developed a hematological malignancy; although pre-B ALL was not the main neoplastic phenotype, two cases diagnosed with this disease were documented (2) (**Supplementary Table 6**).

In terms of cancer predisposition, the unadjusted OR estimates for pre–B ALL in carriers of the *CHEK2* p.Leu236Pro variant, using both the Indigenous Mexican subset and the full MCPS dataset as control groups (**Supplementary Table 3**) are imprecise, reflecting the limited number of affected individuals. The small sample size reduces the precision of risk estimation and limits prognostic inference. However, quantitative bias analysis and E-value calculations suggest that the observed association is robust even when accounting for potential residual confounding factors such as age and sex, as demonstrated by sensitivity analyses.

These findings support a consistent association between the *CHEK2* p.Leu236Pro variant and predisposition to pre-B ALL. However, it cannot be interpreted as the cause of the disease, given the inherent limitations of the study design. Therefore, the results of this study should be interpreted as preliminary evidence, which warrants replication in larger cohorts in order to more accurately define the magnitude of risk conferred by the *CHEK2* p.Leu236Pro variant in this disease.

Flores-Lagunes et al. (26) described the cancer spectrum associated with the p.Leu236Pro variant in Mexican adults. The spectrum predominantly includes breast cancer (67.6%), followed by ovarian cancer (8.1%), prostate cancer (6.7%), gastric cancer (4.1%), thyroid cancer (2.7%), and endometrial cancer (2.7%) (24). Interestingly, pre-B ALL was not included in the cancer spectrum associated with the p.Leu236Pro variant in this study, possibly because it did not focus on the pediatric population. Nevertheless, the occurrence of cancer in the ALL001 patient’s family (**Figure 1**) is consistent with the *CHEK2-*cancer predisposition spectrum, which includes breast, uterine, and skin cancers.

A *CHEK2*-cancer predisposition spectrum was also observed in other pre-B ALL patients who were positive for *CHEK2* GVs. For instance, Ipe et al. (19) reported a male with pre-B ALL and the *CRLF2::P2RY8* fusion who also carried the *CHEK2* p.Thr367Metfs*15 variant. Seven months after the pre-B ALL diagnosis, he was diagnosed with papillary thyroid cancer. His family history revealed that his maternal uncle died of metastatic thyroid cancer at 44 years of age (19). Another case, described by Junk et al. (17), involved a 16-month-old male diagnosed with hyperdiploid pre-B ALL who presented with a *CHEK2* p.Arg117Gly variant. Two years and three months after his pre-B ALL diagnosis, he was diagnosed with acute myeloid leukemia as a second primary malignancy (17). These cases demonstrate that individuals carrying *CHEK2* GVs may present with pre-B ALL as their primary cancer.

The presence of *CHEK2* GVs in children diagnosed with pre-B ALL can affect conventional therapies. For patients who will receive an allogenic hematopoietic stem cell transplant, identifying relatives who are carriers of deleterious germline variants is critical to avoid selecting a donor who carries *CHEK2* deleterious GVs. Additionally, pediatric patients with pre-B ALL positive for deleterious GVs in DNA-damage response genes require special attention. These patients manifest changes in CHK2 function, which can modify their response to DNA-damage chemotherapeutic drugs. Since a therapy-related toxicity was documented in the ALL001 patient, it is important to consider that a delicate balance between therapeutic efficacy and toxicity is required for conventional chemotherapy in these patients (25, 27).

According to the ACMG clinical management guidelines, heterozygous carriers of missense *CHEK2* deleterious GVs usually have a lower overall cancer risk and penetrance than carriers of truncating variants (27). However, there are some exceptions, such as the *CHEK2* p.Arg117Gly and p.Ile157Thr variants, which are associated with breast cancer in a manner similar to truncating mutations. These exceptions suggest that the penetrance of *CHEK2* missense variants depends on the protein residue involved. Notably, most *CHEK2* deleterious GVs observed in patients with pre-B ALL involve highly conserved residues that potentially affect the function and structure of the CHK2 protein (**Supplementary Table 6**).

Regarding the pathogenicity of the *CHEK2* p.Leu236Pro variant, Delimitsou et al. (23) validated the deleterious impact of the aminoacid substitution using yeast-based models (23). In addition, we proposed a molecular mechanism of pathogenicity, the protein modeling analysis presented here suggests that the mutation can interfere with CHK2 protomer dimerization due to steric hindrance and destabilization of the molecular interactions mediated by residues neighboring Leu236. This could reduce normal protein function, and it is in line with the experimental characterization of this variant (23), Nevertheless, exploring the functional impact of the variant in an experimental model of pre-B ALL is still pending to confirm its involvement in the pathological processes associated with the disease.

Based on our findings and those reported in the literature, *CHEK2* may play a role in the germline origin of childhood pre-B ALL in specific populations. The ethnic origin of the population may be important, particularly in populations where founder mutations are suspected, as the prevalence of *CHEK2* GVs in children with pre-B ALL may depend on it. This study provides preliminary evidence of the presence of *CHEK2* GVs in Mexican children with pre-B ALL. This evidence warrants replication in a larger cohort to obtain a definitive prevalence and to estimate an accurate pre-B ALL predisposition risk.

## Data Availability

The raw data supporting the conclusions of this article will be made available by the authors, without undue reservation.
