# Supplementary material for: CHEK2 germline variants in B-cell precursor acute lymphoblastic leukemia: findings in Mexican pediatric patients
Source: Front Oncol. 2026 Mar 16;16:1751793. doi: 10.3389/fonc.2026.1751793 (PMC13034570; doi:10.3389/fonc.2026.1751793)
Supplement: Supplementary file 1 [file DataSheet1.docx]

*CHEK2* Germline Variants in B-Cell Precursor Acute Lymphoblastic Leukemia: Findings in Mexican Pediatric Patients

Supplementary information.

Supplementary Table 1: Forty-two cancer predisposition genes of the targeted exome sequencing panel…………………………………………..2

Supplementary Table 2. Summary of genetic and clinical features of the *CHEK2* GVs carriers in the present study……………………………….4

Supplementary figure 1. *CHEK2* germline variants analysis…………………………………………………………………………………………6

Supplementary Table 3. Frequencies of *CHEK2* GVs in the cases of the present study and in control carriers, and a comparative analysis of *CHEK2*p.Leu236Pro between cases and control carriers……………………………………………………………………………………………..8

Supplementary methods………………………………..……………………………………………………………………………………………..8

Supplementary Table 4. Quantitative bias analysis…………………………………………………………………………………………………..9

Supplementary Table 5. E-value calculations………………………………………………………………………………………………………..10

Supplementary Table 6. Summary of *CHEK2* germline mutations reported in patients with preB-ALL……………………………………………11

| Supplementary Table 1. Forty-two cancer predisposition genes of the targeted exome sequencing panel | | |
| --- | --- | --- |
| Gene | **Condition** | **Inheritance** |
| *ATM* | Ataxia-telangiectasia | AR |
| *BLM* | Bloom syndrome | AR |
| *BRCA1* | Familial breast-ovarian cancer, pancreatic cancer susceptibility.  Fanconi anemia | AD  AR |
| *BRCA2* | Familial breast-ovarian cancer, pancreatic cancer susceptibility.  Fanconi anemia | AD  AR |
| *BRIP1* | Breast cancer susceptibility  Fanconi anemia | AD  AR |
| *CEBPA* | Familial acute myeloid leukemia | AD |
| *ETV6* | ALL predisposition | AD |
| *FANCA* | Fanconi anemia | AR |
| *FANCC* | Fanconi anemia | AR |
| *FANCE* | Fanconi anemia | AR |
| *FANCF* | Fanconi anemia | AR |
| *FANCG* | Fanconi anemia | AR |
| *GAB2* | Putative ALL predisposition | AD |
| *GATA2* | MDS/AML susceptibility | AD |
| *IKZF1* | CVID, ALL predisposition | AD |
| *MSH2* | Lynch syndrome  CMMRD | AD  AR |
| *MSH6* | Lynch syndrome  CMMRD | AD  AR |
| *MYH9* | Macrothrombocytopenia and granulocyte inclusions with or without nephritis or sensorineural hearing loss | AD |
| *NBN* | Breast cancer susceptibility  Nijmegen breakage syndrome | AD  AR |
| *NF1* | Neurofibromatosis type I | AD |
| *PAX5* | ALL susceptibility | AD |
| *PTPN11* | Noonan syndrome | AD |
| *RAD51C* | Breast-ovarian cancer susceptibility  Fanconi anemia | AD  AR |
| *RUNX1* | Familial platelet disorder with associated myeloid malignancy | AD |
| *SH2B3* | ALL and MPN susceptibility | AR |
| *TET2* | Myeloid malignancies and lymphoma susceptibility | AD |
| *TP53* | Li-Fraumeni syndrome | AD |
| *TYK2* | Putative ALL predisposition | AD |
| *APC* | Adenomatous polyposis coli | AD |
| *ATR* | Familial cutaneous telangiectasia and cancer syndrome; Seckel syndrome | AD  AR |
| *CDH1* | Familial gastric carcinoma | AD |
| *CDKN1B* | Multiple endocrine neoplasia | AD |
| *CDKN2A* | Familial malignant melanoma | AD |
| *CHEK2* | Familial breast cancer | AD |
| *CXCR4* | WHIM syndrome | AD |
| *EGFR* | Lung cancer susceptibility | AR |
| *ERCC2* | Xeroderma pigmentosum | AR |
| *KIT* | Familial gastrointestinal stromal tumor | AD |
| *PDGFRA* | Familial gastrointestinal stromal tumor | AD |
| *RB1* | Familial retinoblastoma | AD |
| *WRN* | Werner syndrome | AR |
| *AXIN2* | Oligodontia-colorectal cancer syndrome | AD |
| AR: Autosomal recessive. AD: Autosomal dominant. | | |

| Supplementary Table 2. Summary of genetic and clinical features of the *CHEK2* GVs carriers in the present study | | | | |
| --- | --- | --- | --- | --- |
| Patient ID | **ALL001** | **ALL002** | **ALL003** | **ALL004** |
| *CHEK2* variant | c.836T>C  (p.Leu236Pro) | c.836T>C  (p.Leu236Pro) | c.275C>A  (p.Pro92His) | c.434G>A  (p.Arg145Gln) |
| VAF | 0.526868 | 0.491448 | 0,4976 | 0,5074 |
| CNVs in *CHEK2*  genomic region | Negative | Negative | Negative | Negative |
| ACMG classification | LP  PS4,PS3,PM2 | LP  PS4,PS3,PM2 | VUS  PM2 | VUS  PM2,PM5,PP3 |
| ClinVar status and identifier | Germline LP  #142448 | Germline LP  #142448 | Germline VUS  #479592 | Germline VUS  #141337 |
| SNP identifier | rs587782471 | rs587782471 | rs779269031 | rs587781667 |
| Germline validation | Positive | n.a | Positive | n.a |
| Inheritance | Maternal | n.a | n.a | n.a |
| Other pathogenic germline variants | Negative | *PTPN11* c.172A>T  (p.Asn58Tyr) | Negative | Negative |
| Gender | Female | Male | Female | Female |
| Age at diagnosis (years) | 13 | 14 | 8 | 16 |
| Hemoglobin (g/dL) | 11.2 | 9.5 | 7.3 | 6.4 |
| Hematocrit (%) | 47.2 | n.a | 24.1% | n.a |
| Platelets (10^3^/µL) | 11 | 6 | 3 | 26 |
| Blast percentage in bone marrow | 86% | 93% | 90% | 99% |
| WBC count at diagnosis (x10^3^/ µL) | 467.700 | 400 | 174.600 | 34.700 |
| Immunophenotype | Pre-B  HLDR 55%, CD10 6%, CD19 99%, CD20 77%, CD34 13%, CD79 78% | Pre-B  CD10 92%, CD19 97%, CD20 1%, CD34 70%, CD79 10%. | Pre-B  HLDR 92%, CD10 99%, CD19 96%, CD20 66%, CD34 39%, CD79 90%. | Pro-B  CD10 2.1%, CD19 66.6 %, CD20 0.8%, CD34 98%, CD79 57 % |
| preB-ALL genetic subtype | Ph-like  (*CRLF2::IGH)* | B-Other | B-Other | B-Other |
| NCI Risk | High | High | High | High |
| Extramedullary infiltration | Positive | n.a | Negative | n.a |
| Chemotherapeutic treatment | BFM90  COG AALL1331 | n.a | StJude XII B | n.a |
| Bone marrow relapse | Positive | n.a | Negative | n.a |
| Death | Positive | Positive | Positive | n.a |
| Familiar history of cancer | Positive | Negative | Negative | Positive |
| LP: Likely pathogenic. VUS: Variant of uncertain significance n.a: not available. Ph-like: Philadelphia chromosome-like. B-Other: Genetically unclassified disease. | | | | |

**
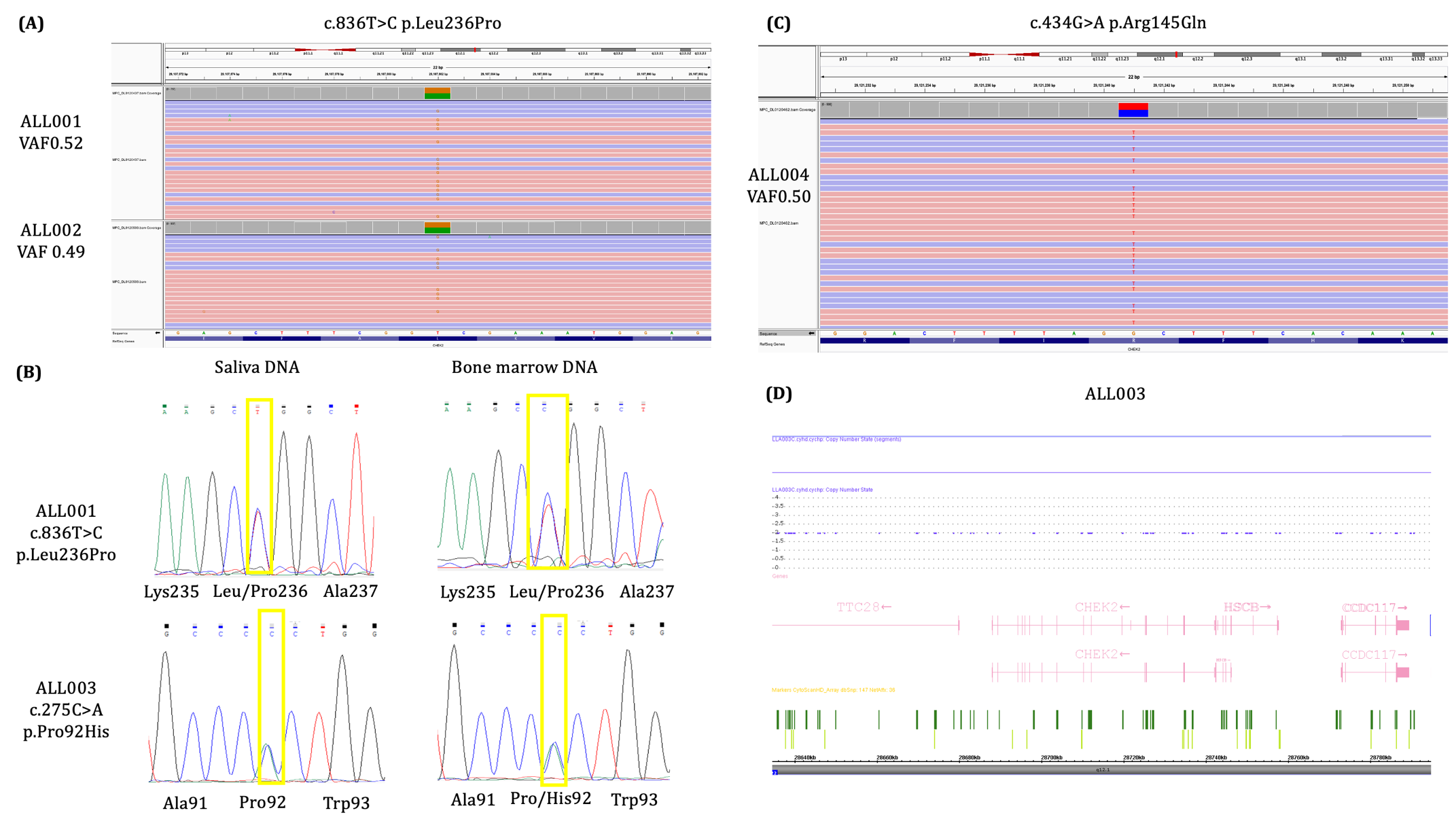
**

**Supplementary Figure 1.** *CHEK2* germline variants analysis. (A) IGV screenshot showing the *CHEK2*p.Leu236Pro mutation detected by NGS reads.  (B) Germinality confirmation of *CHEK2* variants in patients ALL001 and ALL003. (C) IGV screenshot showing the *CHEK2*p.Arg145Gln mutation detected by NGS reads.   (C) ChAS software screenshot of a representative patient showing the normal copy number status of *CHEK2.*

| Supplementary Table 3. Frequencies of *CHEK2* GVs in the cases of the present study and in control carriers, and a comparative analysis of *CHEK2*p.Leu236Pro between cases and control carriers | | | | | | | | | |
| --- | --- | --- | --- | --- | --- | --- | --- | --- | --- |
| *CHEK2* variant | **Carriers’ cases** | **Total cases** | **Frequency** | **Carriers’ controls** | **Total**  **controls** | **Frequency** | **Unadjusted OR** | **95% IC** | ***P* value** |
| c.836T>C (p.Leu236Pro) | 2 | 73 | 2.7% | 87 ^G^  961 ^A^  961 ^IMX^ | 251,368 ^G^  276,400^A^  187,956 ^IMX^ | 0.03476%  0.0346%  0.05112% | 8.07  5.48 | 1.97-32.96  1.34-22.37 | <0.001  0.027  0.054* |
| c.434G>A (p.Arg145Gln) | 1 | 73 | 1.3% | 4 ^A^  0 ^IMX^ | 276,400 ^A^  187,956 ^IMX^ | 0.0015%  0% | n.c | n.c | n.c |
| c.275C>A (p.Pro92His) | 1 | 73 | 1.3% | 0 | 0 | 0% | n.c | n.c | n.c |
| G: GnomAD exomes. A: All patients of MCPS. IMX: Indigenous Mexican subset of MCPS. * Value with tendency towards statistical significance n.c: non calculated. | | | | | | | | | |

Supplementary methods.

Quantitative bias analysis and E-value calculations: Given the germline nature of the condition, age and sex were considered the only plausible sources of unmeasured confounding factors. These were modelled together as a single hypothetical confounder to capture differences in demographics and selection between pediatric cases and the adult control population. Plausible ranges for confounder prevalence among controls were specified under conservative assumptions. Relative risks of 2, 5, and 10 were specified to represent moderate, strong, and extreme confounding scenarios, respectively. These values were used solely as sensitivity parameters and were not intended to represent specific measured confounders. Bias-adjusted ORs were obtained by dividing the unadjusted OR by the corresponding bias factor.

E-values were calculated according to the method proposed by VanderWeele and Ding (2017) using the formula *E = OR + √[OR × (OR − 1)]* for both the unadjusted ORs and the lower bounds of the 95% confidence intervals.

| **Supplementary Table 4. Quantitative Bias Analysis** | | | | | | |
| --- | --- | --- | --- | --- | --- | --- |
| Scenario | Confounder | Assumed RR | P (Cases) | P(Controls)**^+^** | Bias factor* | Bias-adjusted OR |
| Unadjusted OR (Our cohort vs MCPS-ALL): 8.07 | | | | | | |
| 1 | Sex (female) | 2 | 0.575 | 0.50 | 1.05 | 7.69 |
| 2 | Sex (female) | 5 | 0.575 | 0.50 | 1.10 | 7.34 |
| 3 | Sex (female) | 10 | 0.575 | 0.50 | 1.15 | 7.02 |
| 4 | Age ≥10 years | 2 | 0.342 | 0.30 | 1.03 | 7.83 |
| 5 | Age ≥10 years | 5 | 0.342 | 0.30 | 1.08 | 7.49 |
| 6 | Age ≥10 years | 10 | 0.342 | 0.30 | 1.15 | 7.02 |
| 7 | Sex+Age ^**^ | 5+5 | n.a | n.a | 1.19 | 6.81 |
| 8 | Sex+Age^**^ | 10+10 | n.a | n.a | 1.33 | 6.07 |
| Unadjusted OR (Our cohort vs MCPS-IMX): 5.48 | | | | | | |
| 1 | Sex (female) | 2 | 0.575 | 0.50 | 1.05 | 5.22 |
| 2 | Sex (female) | 5 | 0.575 | 0.50 | 1.10 | 4.98 |
| 3 | Sex (female) | 10 | 0.575 | 0.50 | 1.15 | 4.77 |
| 4 | Age ≥10 years | 2 | 0.342 | 0.30 | 1.03 | 5.32 |
| 5 | Age ≥10 years | 5 | 0.342 | 0.30 | 1.08 | 5.09 |
| 6 | Age ≥10 years | 10 | 0.342 | 0.30 | 1.15 | 4.77 |
| 7 | Sex+Age^**^ | 5+5 | n.a | n.a | 1.19 | 4.62 |
| 8 | Sex+Age^**^ | 10+10 | n.a | n.a | 1.33 | 4.12 |
| RR: Relative risks. P: Prevalence. **^+^** Control prevalences were specified under conservative scenarios. *Bias factors were calculated following the method of Lash et al., (2009). ^**^Combined bias factors assume independence between age and sex. n.a: Not applicable for combined confounder scenarios. | | | | | | |

| **Supplementary Table 5. E-value calculations** | | | | |
| --- | --- | --- | --- | --- |
| Control reference | Unadjusted OR | 95% Confidence Interval | E value for unadjusted OR | E value for lower confidence Interval |
| MCPS-ALL | 8.07 | 1.97-32.96 | 15.62 | 3.35 |
| MCPS-IMX | 5.48 | 1.34-22.37 | 10.43 | 2.01 |

IMX: Indigenous Mexicans.

| **Supplementary Table 6. Summary of *CHEK2* germline mutations reported in patients with preB-ALL** | | | | | | | | | | | | | |
| --- | --- | --- | --- | --- | --- | --- | --- | --- | --- | --- | --- | --- | --- |
| Case ID ^(Reference)^ | Gender | Age of diagnosis (years) | preB-ALL molecular subtype | Familial history of cancer | Inheritance | Origin population | Population frequency  (gnomAD exomes) | *CHEK2*  GVs | ACMG/AMP Classification | Pathogenicity prediction | Protein stability change | Conservation analysis | Functional characterization  ^(Reference)^ |
| P2  ^(13)^ | Male | 12 | *PAX5::*  *SOX5* | MGF: Pancreas cancer (<45yrs) | Paternal | n.a | 0.0091% | c.444+1G>A | Pathogenic PM3,PS3,PVS1,PM2 | n.a | n.a | n.a | Damaging  ^(19)^ |
| SJHYPO00  ^(14)^ | n.a | <20 | Hypodiploid | n.a | n.a | n.a | 0.002% | c.134C>T (p.Thr45Met) | VUS  PM2, BP4 | Damaging  (2/3) | Decreased  (2/2) | Variable  (2/2) | n.a |
| ALL003  ^(This study)^ | Female | 8 | B-Other | Negative | n.a | Mexican | 0% | c.275C>A  (p.Pro92His) | VUS  PM2 | Damaging  (2/3) | Decreased  (2/2) | Variable  (2/2) | n.a |
| #230  ^(15)^ | Male | 1.6 | Hyperdiploid | n.a | n.a | Germany | 0.01119% | c.349A>G (p.Arg117Gly) | Pathogenic PS4,PVS1,PM2 | Damaging  (3/3) | Decreased  (2/2) | Conserved  (2/2) | Damaging  ^(20)^  Benign  ^(19)^ |
| #307  ^(15)^ | Female | 3.6 | n.a | n.a | n.a |  |  |  |  |  |  |  |  |
| SJPHALL020047  ^(15)^ | n.a | <20 | *BCR::ABL1* | n.a | n.a | n.a | 0.0024% | c.409C>T (p.Arg137*) | Pathogenic PVS1,PM2,PS4,PP5 | Damaging  (1/1) | n.a | Average  (2/2) | n.a |
| ALL004  ^(This study)^ | Female | 16 | B-Other | Breast and Gastric cancer in Paternal relatives. | n.a | Mexican | 0.00000072 % | c.434G>A (p.Arg145Gln) | VUS  PM2 PM5,PP3 | Damaging  (2/3) | Decreased  (2/2) | Average  (2/2) | Benign  ^(20,21)^  Damaging  ^(22)^ |
| D4  ^(13)^ | Female | 3 | n.a | Negative | Maternal | Germany | 0.4121% | c.470T>C (p.Ile157Thr) | Likely Pathogenic PS4,PM2,PM5 | Damaging  (2/3) | Decreased  (2/2) | Average  (2/2) | Damaging, Intermediate, Benign  ^(20)^ |
| P8  ^(13)^ | Female | 12 | n.a | Negative | Maternal | Germany |  |  |  |  |  |  |  |
| P10  ^(13)^ | Female | 3 | n.a | Negative | Paternal | Germany |  |  |  |  |  |  |  |
| P12  ^(13)^ | Female | 2 | n.a | Negative | Paternal | Germany |  |  |  |  |  |  |  |
| #5591  Two cases  ^(2)^ |  | n.a | n.a | n.a | n.a | Western European (Polish) |  |  |  |  |  |  |  |
| P13  ^(13)^ | Male | 10 | n.a | Negative | Maternal | Germany | 0.0019% | c.480ª>G  (p.Ile160Met) | VUS  PM2,PP3 | Damaging  (3/3) | Decreased  (2/2) | Conserved  (2/2) | Damaging  ^(20)^  Benign  ^(13,20)^ |
| P14  ^(13)^ | Male | 2 | n.a | Negative | Paternal | Germany |  |  |  |  |  |  |  |
| P16  ^(13)^ | Male | 7 | n.a | Negative | Paternal | Germany |  |  |  |  |  |  |  |
| P17  ^(13)^ | Female | 1 | Normal karyotype | MGM: Blood malignancy (34yrs) | Paternal | Germany |  | c.499G>C  (p.Gly167Arg) | Pathogenic  PS1,PP3,PM2,PP5 | Damaging  (3/3) | Decreased  (2/2) | Conserved  (2/2) | Damaging  ^(13, 20)^ |
| SJPHALL020036  ^(15)^ | n.a | <20 | *BCR::ABL1* | n.a | n.a | n.a | 0.0111% | c.541C>T (p.Arg181Cys) | Pathogenic PM2,PM1PM5,PP5,BP6 | Benign (2/3) | Decreased  (2/2) | Average (2/2) | Damaging  ^(13, 20)^ |
| D5  ^(13)^ | Female | 5 | n.a | MGM: Bone cancer, cervical cancer (<45yrs) | Paternal | Germany |  |  |  |  |  |  |  |
| ALL001  ^(This study)^ | Female | 13 | *CRLF2::*  *IGH* | MMR: cerebral cancer, uterine cancer (37yrs)  skin cancer,  hepatic cancer (5yrs) | Maternal | Mexican | 0.00252% | c.836T>C (p.Leu236Pro) | Likely Pathogenic PS4,PS3,PM2 | Damaging (3/3) | Decreased  (2/2) | Conserved  (2/2) | Damaging  ^(20,21)^ |
| ALL002  ^(This study)^ | Male  13 years |  | B-Other | n.a | n.a | Mexican |  |  |  |  |  |  |  |
| SJERG022  ^(13)^ | n.a | <20 | *ERG* altered | n.a | n.a | n.a |  |  |  |  |  |  |  |
| SJHYPO041  ^(14)^ | n.a | <20 | Hypodiploid | n.a | n.a | n.a | 0.0076% | c.715G>A (p.Glu239Lys) | VUS  PM2 | Damaging  (2/3) | Decreased  (2/2) | Conserved  (2/2) | Damaging, Intermediate, Benign  ^(20)^ |
| SJETV041  ^(14)^ | n.a | <20 | *ETV6::*  *RUNX1* | n.a | n.a | n.a | 0.0008% | c.818ª>C (p.Glu273Ala) | VUS  PM2, PP3 | Damaging  (3/3) | Decreased  (2/2) | Conserved  (2/2) | Damaging  ^(20)^ |
| P20  ^(13)^ | Male | 9 | Hyperdiploid | Negative | Paternal | Germany |  | c.903del  (p.Leu301Trpfs*3) | Pathogenic PVS1,PM2,PS4 | Damaging (1/1) | n.a | Conserved  (2/2) | Damaging  ^(13)^ |
| P22  ^(13)^ | Female | 3 | n.a | n.a | Maternal | Germany | 0.0052% | c.1035C>T (p.Arg346Cys) | Pathogenic PS3,PM2,PM5,PS4,PP3,PM1,PP5 | Damaging  (3/3) | Decreased  (2/2) | Conserved  (2/2) | Damaging  ^(20)^ |
| SJALL043850_R1  ^(16)^ | Female | 12.8 | *TCF3::*  *PBX1* | n.a | n.a | n.a |  |  |  |  |  |  |  |
| SJERG014  ^(14)^ | n.a | <20 | *ERG* altered | n.a | n.a | n.a | 0.0088% | c.1053G>T (p.Glu351Asp) | VUS  PM2 | Damaging  (2/3) | Decreased  (1/2) | Conserved  (2/2) | Damaging  ^(20)^ |
| ^(17)^ | Male | 12 | *CRLF2::*  *P2RY8* | MU: Thyroid cancer (44yrs) | Maternal | American  (U.S.A) | 0.2044% | c.1100delC (p.Thr367Metfs*15) | Pathogenic PS4,PVS1,PM2 | Damaging (1/1) | n.a | Conserved  (2/2) | Damaging  ^(13)^ |
| P23  ^(13)^ | Female | 15 | *BCR::*  *ABL1* | MGM:Breast cancer (49yrs) | Maternal | Germany |  |  |  |  |  |  |  |
| SJBALL030247_D1  ^(18)^ | Female | 18.8 | n.a | n.a | n.a | n.a |  |  |  |  |  |  |  |
| SJERG014  ^(14)^ | n.a | <20 | *ERG* altered | n.a | n.a | n.a | 0.0386% | c.1312G>T (p.Asp438Tyr) | VUS  PM2, BP6 | Damaging  (3/3) | Decreased  (2/2) | Conserved  (1/2) | Damaging, Intermediate, Benign  ^(20)^ |
| SJERG020053  ^(14)^ | n.a | <20 | *ERG* altered | n.a | n.a | n.a | Non reported | c.1448ª>G (p.His483Arg) | VUS  PM2, PP3 | Damaging  (2/3) | Decreased  (2/2) | Conserved  (2/2) | Damaging  ^(20)^ |
| SJHYPER062  ^(14)^ | n.a | <20 | n.a | n.a | n.a | n.a | 0.0004% | c.1555C>T (p.Arg519*) | Pathogenic PS4,PS3,PVS1 | Damaging  (1/1) | n.a | Average (2/2) | Intermediate  ^(20)^ |
| MGF: Maternal grandfather. MGM: Maternal grandmother MU:Maternal uncle. MMR:Multiple maternal relatives. n.a: not available. Yrs: Years. B-Other: Genetically unclassified disease. GVs: Germline variants | | | | | | | | | | | | | |
